# The COVID-19 pandemic and its implications for the food information environment in Brazil

**DOI:** 10.1017/S1368980020004747

**Published:** 2020-11-23

**Authors:** Michele Bittencourt Rodrigues, Juliana de Paula Matos, Paula Martins Horta

**Affiliations:** Departamento de Nutrição, Universidade Federal de Minas Gerais, Av. Alfredo Balena 190, Escola de Enfermagem, 3º andar, sala 312, Belo Horizonte, 30130-100 Minas Gerais, Brazil

**Keywords:** Food information environment, COVID-19 pandemic, Marketing strategies, Food industry, Ultra-processed foods

## Abstract

The food information environment includes food advertising disseminated in various media. With the COVID-19 pandemic and the shutdown of schools, universities, non-essential commerce, public leisure areas, bars, restaurants, among others, the food information environment has changed in Brazil. People spent more time at home which led to greater exposure to television and internet advertising content. During the COVID-19 pandemic, the food production sector has invested in new ways to advertise their products that include advertising messages of support, empathy and solidarity, as well as social responsibility campaigns looking for self-promotion such as food donation and financial aids. Sponsoring online events promoted by Brazilian musicians on social media was also enhanced during the pandemic and allowed food companies to become part of the consumer’s leisure and entertainment moments. The advertising strategies adopted by the food industry during the COVID-19 pandemic are used to generate market demands, influence the consumer purchase decision and increase their loyalty to the supplier brands. Consequently, individuals may have been more vulnerable to excessive consumption of ultra-processed foods during this health crisis. This commentary aims to describe the changes in the food information environment during the COVID-19 pandemic in Brazil and propose a pathway to promote a healthier food information environment after this health crisis. Perspectives for promoting a healthier food information environment after the pandemic are also discussed, focusing on regulating food advertising with a shared responsibility between government, the food industry, the academy and civil society.

COVID-19 is the disease caused by the new coronavirus,SARS-CoV-2, which was first reported in China in December 2019^(1)^.Subsequently, on 11 March 2020, the WHO declared COVID-19 as a pandemic^(2)^. In Brazil, the first case of the disease was confirmed on 26 February 2020^(3)^.

In an attempt to prevent and slow the spread of the virus, some social distancing practices were implemented^(4)^ that resulted in the shutdown of schools, universities, non-essential commerce, public leisure areas, bars, restaurants, among others. Consequently, people spent more time at home which led to prolonged exposure to television and internet advertising content. The time people spend watching television increased by 1 h and 20 min^(5)^, and the internet usage grew up by 40–50 % during the pandemic^(6)^. Furthermore, people have also changed their work habits and the way they consume goods and how they spend their time off^(4)^, which impacted on how the food industry operates.

All these changes have had an impact on the Brazilian food information environment, which is characterised by food advertising through various media^(7,8)^. Systematic reviews and meta-analysis showed an association between excessive exposure to food advertising and unhealthy eating^(9,10)^. The contextual changes resulting from the COVID-19 pandemic may have increased the population exposure to ultra-processed food (UPF) advertising, which can have a negative impact on consumers’ health.

The objective of this commentary is to describe food information environment changes during the COVID-19 pandemic in Brazil and propose a pathway to promote a healthier food information environment after this health crisis.

## Food industry advertising during the COVID-19 pandemic

Monitoring studies conducted before the COVID-19 pandemic already pointed to a high prevalence of UPF advertising on television^(11)^, social media^(12)^ and food delivery apps^(13)^. During the current health crisis, new marketing strategies have emerged.

**Fig. 1:**
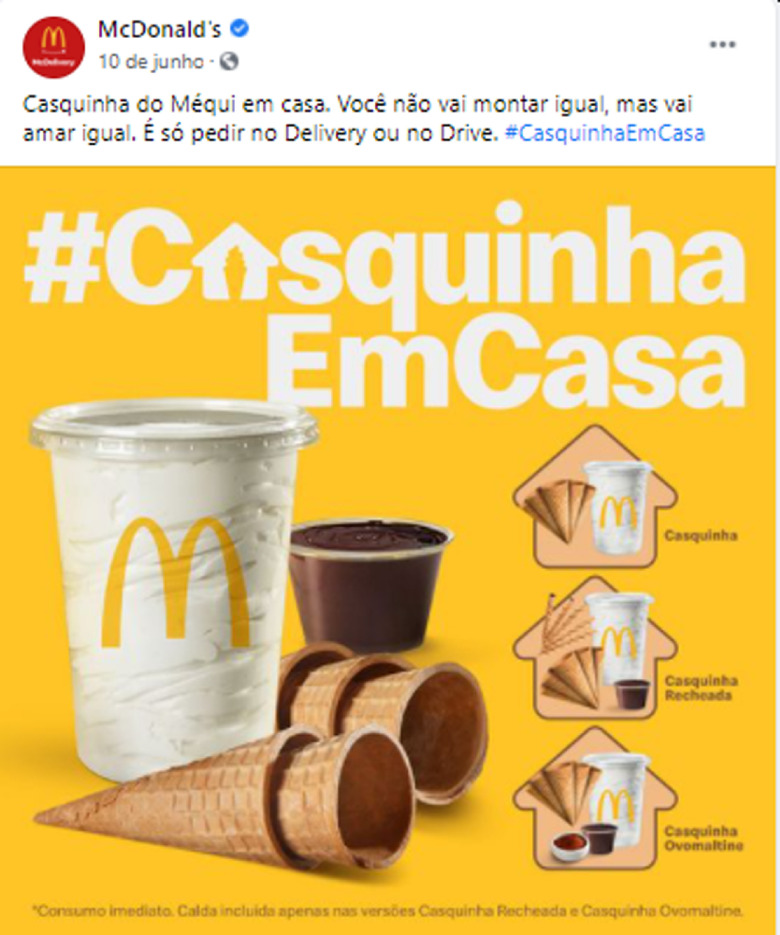
Facebook post made by a fast-food company to stimulate food delivery. The text reads: ‘Cornet at home. You won’t dress up the same but will love it the same. Order for delivery or through the drive-through’

The advertisements have been aligned with the context of social distancing, using messages that appeal to eating at home, reinforcing the idea of staying at home with family and stimulating personal interaction through digital technologies such as video calls. An example is the fast-food industry sharing advertising messages to stimulate food delivery (Fig. 1; see online supplementary material, Supplemental Figures 1 and 2).

**Fig. 2:**
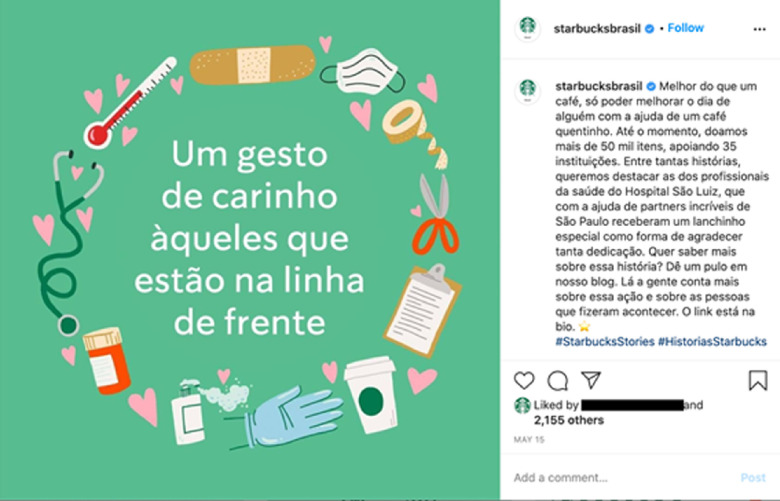
Facebook post made by a chain of coffeehouses advertising donations for health professionals. The text reads: ‘Better than a coffee is to make someone else’s day better with a warm coffee’. ‘So far, we have donated more than 50 thousand items which helped 35 institutions’. ‘A gesture of affection towards the professionals who are in the front line’

Food companies, especially ultra-processed ones, have also carried out social responsibility actions which were identified in campaigns to donate food products, money and personal hygiene items. In general, donations targeted hospitals and philanthropic institutions, health professionals and socially vulnerable populations. These actions gained traction due to the consequences of the economic crisis resulting from COVID-19 in Brazil^(14)^ (Fig. 2).

Another strategy is the promotion of good hygiene practices and protection measures. Examples include big players in the beverage industry redesigning their product labels to display information on how to prevent the spread of the virus (see online supplementary material, Supplemental Figure 3).

Food companies have also highlighted the protective measures they adopted to prevent the infection of their employees. These measures, however, despite being considered essential in the current health context, have been used for self-promotion. An example is food delivery apps which started implementing basic protective measures to their riders (such as offering masks and hand sanitisers) only after being publicly criticised due to the awful working conditions their riders were facing (see online supplementary material, Supplemental Figure 4).

Another advertising strategy that has been used by UPF companies during the COVID-19 pandemic is the sponsorship of live concerts promoted by Brazilian musicians on social media. The ten most watched live events during the pandemic that were still available for access on YouTube on 23 July 2020 gathered a total of 174 million views^(15)^. In these events, UPF companies can be identified by merchandising actions, by the direct consumption or the indication that the product was or will be consumed (e.g., by exposure of the product close to the artists) or finally by explicit advertisements made by the musicians themselves. For exemplification, an ultra-processed meat company developed a website to archive all the events that have been sponsored by the brand along with a channel for buying its products (see online supplementary material, Supplemental Link 1).

All these strategies can negatively impact on consumers’ health. First, social responsibility actions can increase the influence of the UPF industry on the country’s health agendas. Their lobbying has always been present^(16)^, but we have noticed an intensification of this practice during the pandemic^(17)^. Second, the increased exposure of consumers to advertising of UPF may result in greater consumption of this kind of food^(18)^ with unfavourable public health outcomes, such as overweight, obesity and cardiometabolic risks^(19)^.

## Implications of the food information environment in the COVID-19 pandemic for consumer behaviour

The advertising strategies adopted by the food industry during the COVID-19 pandemic are generating market demands at the time of crisis, influencing the consumer purchase decision and increasing their loyalty to the supplier brands. Companies take advantage of a period of increased vulnerability of the population in terms of social, economic and personal relationships to consolidate their market share and reach new consumers.

The advertising messages of empathy and solidarity adopted by the food companies in their advertisements reflect a characteristic of humanisation of the brand that is associated with a closer relationship between company and consumer^(20)^. Consumers’ exposure to brands through the sponsorship of live events means that the food company becomes part of the consumer’s leisure and entertainment moments, encouraging consumers to choose a certain food or drink to consume during the broadcasting in function of what is being advertised. It is also worth mentioning the power of celebrities consuming or endorsing a product to influence the consumer’s choice^(21)^.

These changes in the food information environment, associated with other changes in the individual’s context resulting from social distancing, such as greater exposure to media, new leisure and work habits, changes in the domestic routine, economic recession and changes in mental health, may leave individuals more vulnerable to excessive consumption of UPF^(22,23)^.

An example is the growth of online commerce. During the lockdown, food delivery through apps grew by 9 % on weekdays and 10 % on weekends^(5)^. Previous monitoring studies have demonstrated there is a large offer of UPF in such apps^(13,24)^; thus, a wider use of these tools can result in greater consumption of this food.

The Brazilian online behaviour survey *ConVid*, carried out from April to May 2020 with 45 161 people, registered a decrease of about 4 % in regular consumption (5 d or more a week) of vegetables and beans and an increase of 4 % in frozen meals, 4 % in savoury snacks and 6 % in chocolate/sweet biscuits/pieces of tart from 2 d or more a week^(25)^. In contrast, the *NutriNet Brasil* study, survey conducted with 10 116 individuals, showed a slight increase in the frequency of consumption of vegetables, fruits and beans, as well as a stable high consumption of UPF during the COVID-19 pandemic^(26)^.

In any case, both the increase in the consumption of UPF and their consumption in high frequency could increase the prevalence of overweight (55·4 %) and obesity (20·3 %) in Brazil^(27)^, which can worsen the health condition of individuals with COVID-19 since these conditions are independent risk factors in the prognosis of the disease^(28)^. This demonstrates the need for new ways to minimise the impact of exposure to UPF advertising in Brazil after the pandemic.

## Perspectives for the promotion of a healthy food information environment after the COVID-19 pandemic

Discussing a possible pathway to develop a healthier food information environment after the COVID-19 pandemic involves updating the existing food advertising regulation to the current context, enforcing its compliance and monitoring the food industry practices.

The Brazilian legislation regarding food advertising is described in the Consumer Protection Code (‘Código de Defesa do Consumidor’ – CDC in Portuguese), which guarantees the individual’s right to clear and adequate information about different products and services. The CDC protects the consumer against both misleading advertising, which is capable of inducing the consumer to misinterpret the information about the various aspects of a product, and abusive advertising, that is, the one which is capable of inducing the consumer to behave in a risky manner regarding their health or safety^(29)^. Also, the resolution 163/2014 of the National Council for the Rights of Children and Adolescents (‘Conselho Nacional dos Direitos da Criança e do Adolescente’ – Conanda in Portuguese) handles the abusive advertising targeted to children^(30)^.

Some limits on the application of this legislation have been observed in the digital environment. The wide reach and the many possibilities of engagement with advertising content in this environment allow ordinary people, who are not part of the food industry, to replicate such content by sharing or retweeting it on their social media. Data from the SocialBakers ranking^(31)^ have shown that three of the big UPF companies had alone almost 4 million interactions (shares, likes and comments) on Facebook from April to June 2020. The easy and speedy sharing of these advertisements makes it difficult to trace and attribute responsibility. The process of identifying the advertisements is also hampered by sponsorship strategies involving digital influencers or celebrities. Companies have consistently sponsored these influencers to advertise their products on social media, frequently hiding the commercial partnership behind it.

It is also worth mentioning, as a limitation of the current legislation, the use of personal data from media platforms to track potential consumers. Evidences demonstrate that companies have been using user data available on the internet to target advertisements and influence their choices^(32)^. This affects the consumer’s right to be protected from these practices^(32)^. Unlike General Data Protection Regulation in Europe, until recently Brazil did not have appropriate legislation to regulate data privacy on the internet.

Also, there are no regulations currently in place to protect consumers from the social responsibility actions of the food industry associated with self-promotion or sponsorship. When companies do not take responsibility for the health consequences of consuming their products and divert the attention from questionable practices through corporate social responsibility actions, they end up increasing their credibility before consumers, especially in vulnerable contexts such as the COVID-19 pandemic^(33)^. The health crisis cannot justify the promotion of the unhealthy food industry since it has a crucial impact on the epidemic of obesity and chronic non-communicable diseases, which in turn increases the population’s vulnerability to COVID-19 and emerging infectious diseases^(34,18).^

Finally, it is important to strengthen the regulation of food advertising in Brazil through effective compliance with legislation. This can be achieved by creating channels for registering complaints and forwarding them to the relevant bodies, and also by speeding up the process of dealing with these complaints. The food industry must comply with the regulatory terms in their advertisements and the public authorities must endeavour to inspect their actions. The academy should invest in research on the subject with the aim of systematically monitoring advertising practices in the food industry and their impact on consumer health. Civil society can be motivated through campaigns carried out by the academy and civil society organisations to raise public awareness regarding the issue of food advertising, which aims to encourage their participation in monitoring compliance with legislation by identifying and reporting breaches, and also by policing the competent bodies regarding the forwarding of these.

## Final remarks

The food information environment during the COVID-19 pandemic is changing in Brazil. The food industry, especially those of UPF, has switched their advertising strategy to incorporate messages of empathy, union and partnership, in addition to investing in social responsibility actions and sponsoring online events aiming at self-promotion. As a consequence, the consumer is more vulnerable to the consumption of UPF. Recommendations have been proposed in order to provide a healthier information food environment in Brazil after the COVID-19 pandemic, which include updating the regulatory legislation and its effective compliance through industry accountability and public authority oversight, research on the topic and engagement of civil society.

## Supplementary material

For supplementary material accompanying this paper visit http://dx.doi.org/10.1017/S1368980020004747. click here to view supplementary material
