# Supplementary material for: The COVID-19 pandemic and its implications for the food information environment in Brazil
Source: Public Health Nutr. 2020 Nov 23:1–6. doi: 10.1017/S1368980020004747 (PMC7737163; doi:10.1017/S1368980020004747)

**Supplementary Material – Examples of advertisements from Facebook, Instagram, and Websites in Brazil that reference COVID-19**

**Article: The COVID-19 pandemic and its implications for the food information environment in Brazil**

**Figure 1.** Facebook post made by a fast-food company to stimulate food delivery. The text reads: “If you can stay at home, what are you waiting for to take up the challenge? Staying at home is harsh? It’s simple: more time at home, more delicious products you can earn”.


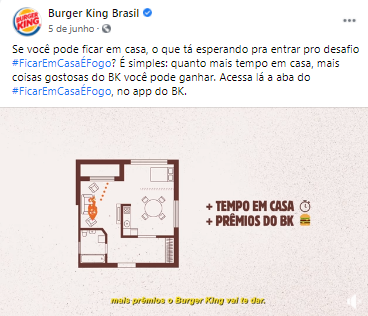


**Figure 2.** Facebook post made by a fast food company to stimulate food delivery. The text reads: “When you are really hungry in your home office. Apparently, now I see pizza everywhere”.


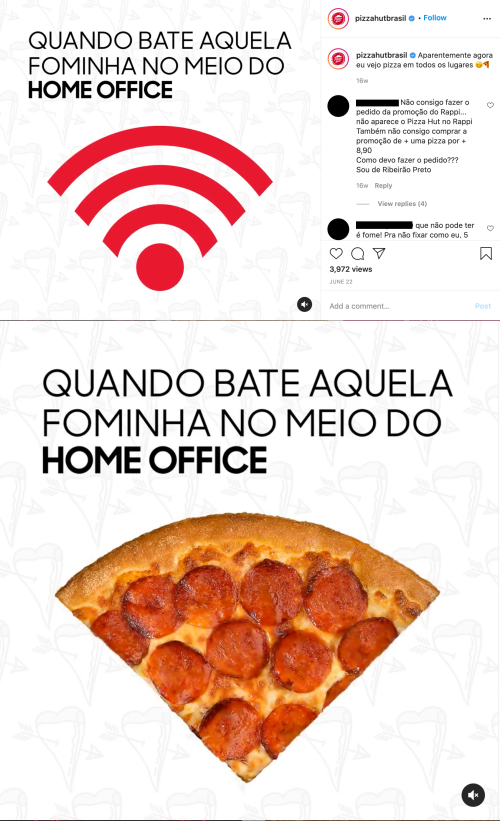


**Figure 3.** Facebook post made by a multinational beverage corporation advertising donation for health professionals. The text reads: “Now Coca-Cola labels will bring information on how to prevent Covid-19. Check it out and put it into practice in your life. Now, every attitude makes a difference. We’ll make it through, together”.


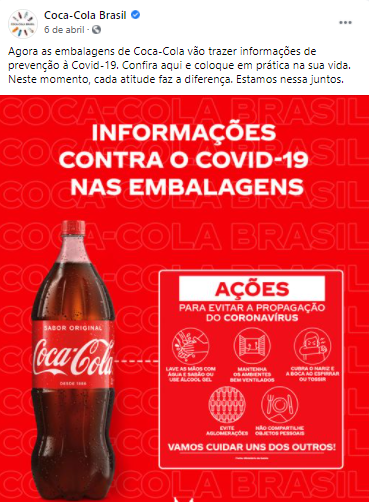


**Figure 4.** Facebook post (video screenshot) made by a food delivery app highlighting their measures to protect their collaborators during the pandemic. They highlight the distribution of hand sanitizers and informative material, as well as the development of a new fund to support the riders. The text invites the users to click on a link to get more information about all the actions they are taking in this regard.


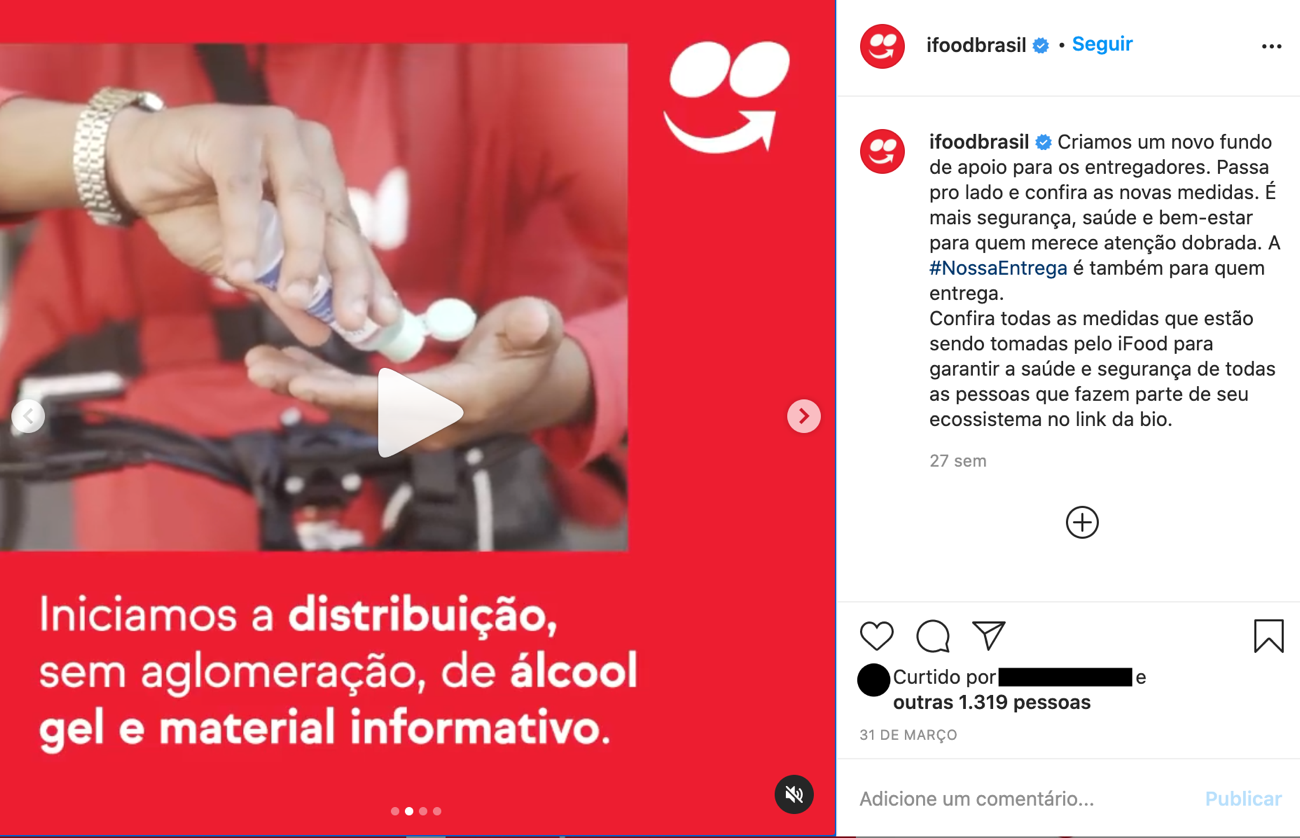


**Link 1.** Link to access an example of action made by an ultra-processed meat company that has been sponsoring live events. They developed a website to organize and archive the events along with a channel for buying its products.

<https://www.searalivefest.com.br/>


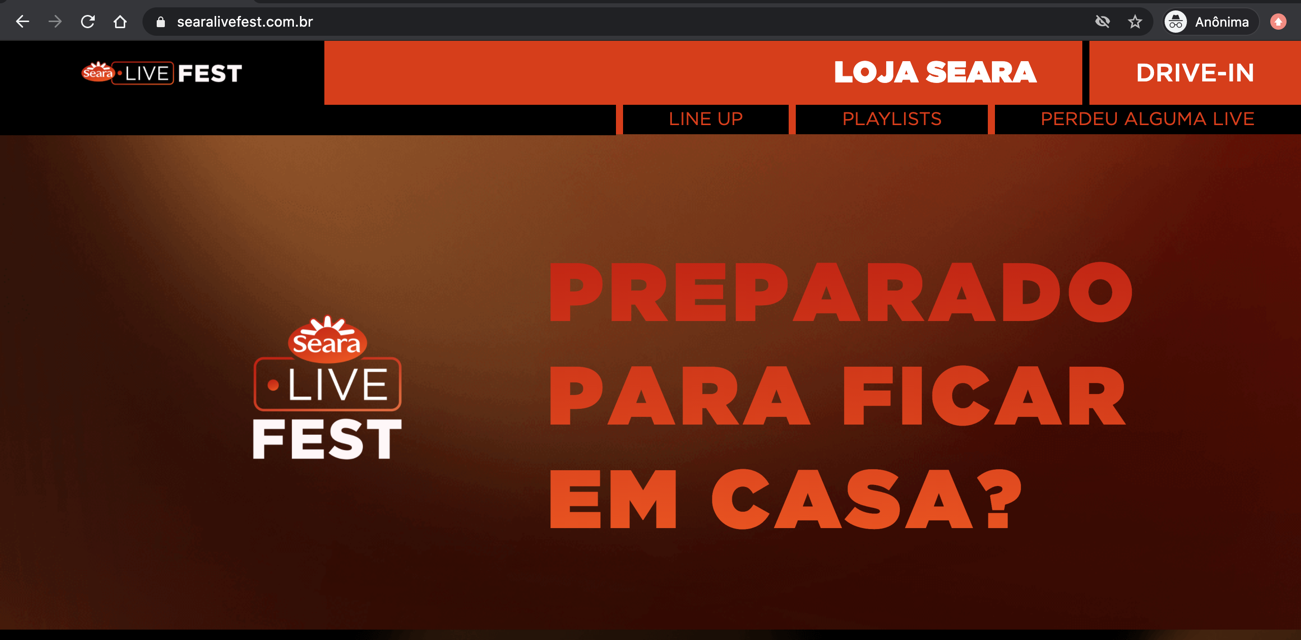

Supplement: Supplementary file 1 [file S1368980020004747sup001.docx]
